# Crystal structure of the complex of 2,4,6-tri­ethyl-1,3,5-tris­[(4-methyl-1*H*-indazol-1-yl)meth­yl]­benzene with NH_4_PF_6_

**DOI:** 10.1107/S2056989022006867

**Published:** 2022-07-12

**Authors:** Felix Fuhrmann, Eric Meier, Wilhelm Seichter, Monika Mazik

**Affiliations:** a Institut für Organische Chemie, Technische Universität Bergakademie Freiberg, Leipziger Str. 29, 09599 Freiberg, Germany; Universidade de Sâo Paulo, Brazil

**Keywords:** crystal structure, tripodal receptor, ammonium complex

## Abstract

The complex of 2,4,6-tri­ethyl-1,3,5-tris­[(4-methyl-1*H*-indazol-1-yl)meth­yl]­benz­ene with ammonium hexa­fluorophosphate, C_39_H_42_N_6_·NH_4_^+^·PF_6_^−^, crystallizes in the monoclinic space group *P*2_1_ with two mol­ecules of the receptor, two NH_4_^+^ and two PF_6_^−^ ions in the asymmetric unit. In each of the complexes the ammonium ion resides in the cavity of the receptor mol­ecule and is fixed in its position by three N—H⋯N bonds, while the remaining hydrogen atom of the cation acts as a bifurcated binding site for N—H⋯F bonding to the counter-anion. The crystal is composed of one-dimensional supra­molecular aggregates extending along the *a*-axis direction.

## Abstract 2

The title complex crystallizes in the monoclinic space group *P*2_1_ with two mol­ecules of the receptor, and two NH_4_^+^ and two PF_6_^−^ ions in the asymmetric unit. In each of the complexes, the ammonium ion resides in the cavity of the receptor mol­ecule and is fixed in its position by three N—H⋯N bonds, while the remaining hydrogen atom of the cation acts as a bifurcated binding site for N—H⋯F bonding to the counter-anion.

## 1. Chemical context

The development of efficient artificial receptors that exhibit high selectivity for ammonium *versus* potassium ions is of great inter­est (Bühlmann *et al.*, 1998 ▸; Chin *et al.*, 1999 ▸; Späth & König, 2010 ▸; Pazik & Skwierawska, 2014 ▸; Jonah *et al.*, 2017 ▸; Schulze *et al.*, 2018 ▸). Both acyclic and macrocyclic receptors have been designed to achieve this goal. Tripodal and hexa­podal benzene derivatives bearing pyrazolyl or indazolyl groups have proven to be promising as receptors for NH_4_^+^ (Chin *et al.*, 1999 ▸, 2002 ▸; Koch *et al.*, 2015 ▸; Jonah *et al.*, 2017 ▸; Schulze *et al.*, 2018 ▸). The ability of these compounds to act as ammonium receptors has been examined both in solution and in the crystalline state. Structural variations are to be used to develop receptor mol­ecules that exhibit a more pronounced selectivity. As part of our studies on structure–binding affinity relationships, we have synthesized various acyclic mol­ecules and investigated their binding properties. In this work we describe the crystal structure of a complex between NH_4_PF_6_ and a tripodal benzene derivative bearing 4-methyl-indazol-1-yl groups. 
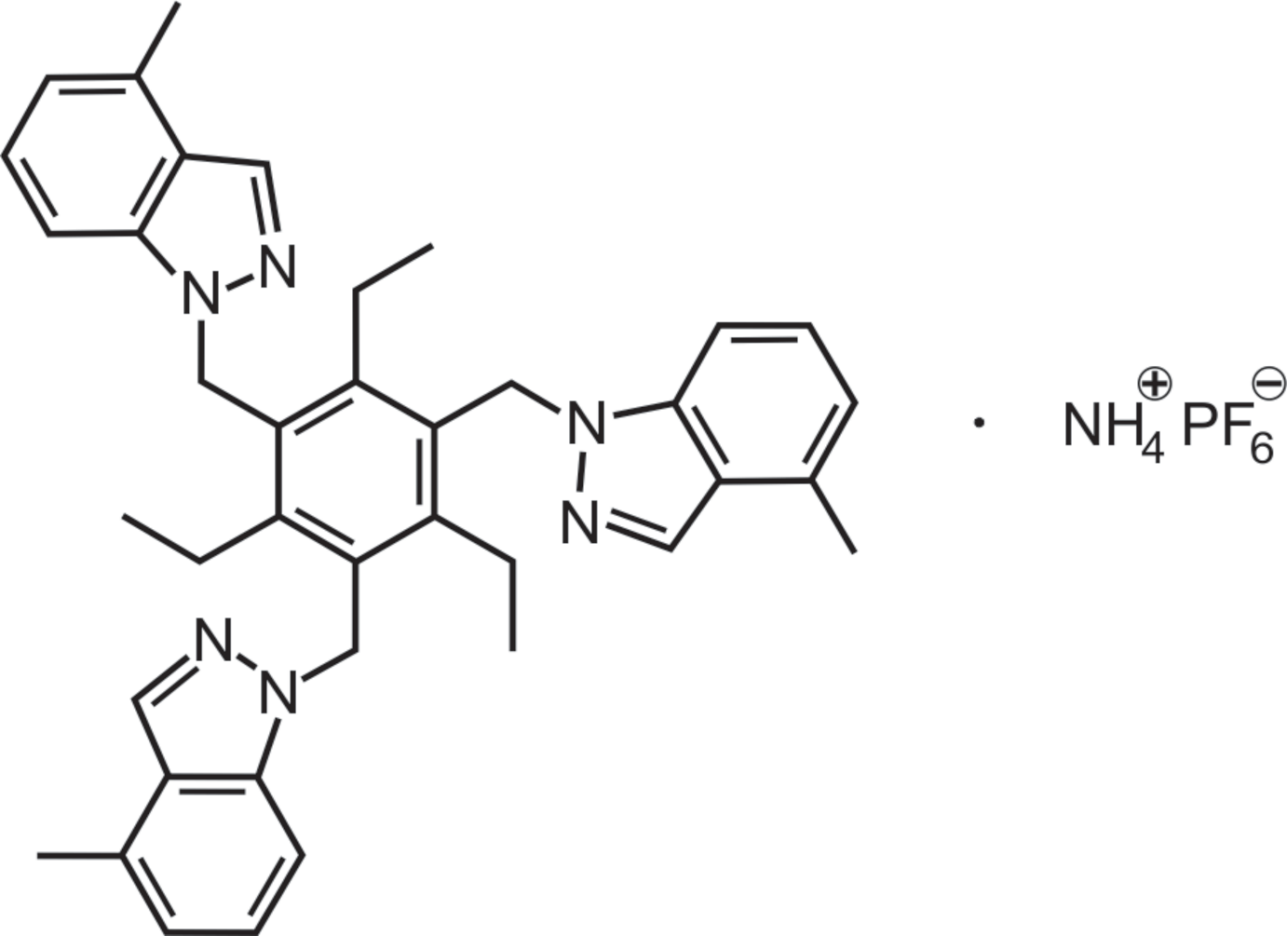


## 2. Structural commentary

**Figure 1:**
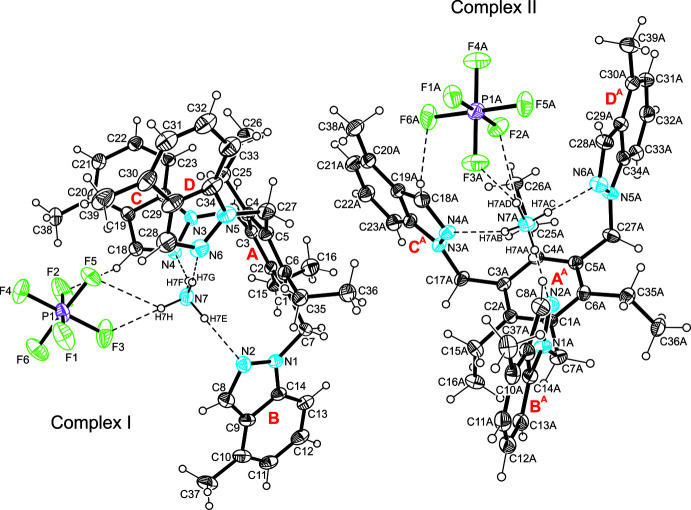
Perspective view of the title complex C_39_H_42_N_6_·NH_4_^+^·PF_6_^−^ including the labelling of non-hydrogen atoms. Displacement ellipsoids are drawn at the 50% probability level. Dashed lines represent hydrogen-bonding inter­actions.

**Figure 2:**
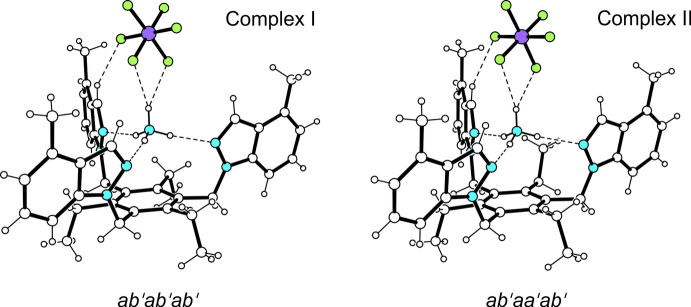
Ball-and-stick representation (side view) of the complexes in the crystal structure of the title complex.

Crystallization from a mixture of the title compound and NH_4_PF_6_ in ethanol yields colourless prisms of the monoclinic space group *P*2_1_ with two mol­ecules of the receptor, two NH_4_^+^ ions and two PF_6_^−^ ions in the asymmetric unit. These components are connected to form complexes of the structures shown in Fig. 1 ▸. In each of them, the NH_4_^+^ ion resides in a cavity created by the indazolyl groups of the receptor and is held in its position by three N—H⋯N bonds [*d*(H⋯N) 2.01 (2)–2.10 (4) Å], involving the nitro­gen atoms designated N2, N4, N6 and N2*A*, N4*A*, N6*A*. The remaining H atom of the ammonium acts as a bifurcated binding site for N—H⋯F bonding with the counter-ion. The conformational difference between the receptor mol­ecules is seen in the orientation of the three ethyl groups, leading to an *ab*′*ab*′*ab*′ (complex I) and an *ab*′*aa*′*ab*′ arrangement (complex II) (*a* = above, *b* = below, *a*′/*b*′ = ethyl; see Koch *et al.*, 2017 ▸; Schulze *et al.*, 2017 ▸) of substituents with respect to the plane of the central arene ring (Fig. 2 ▸). The dihedral angles between the planes of the indazole units are 67.8 (1), 8.1 (2), 72.6 (1)° for complex I and 62.0 (1), 6.9 (2), 65.4 (1)° for complex II.

## 3. Supra­molecular features

**Figure 3:**
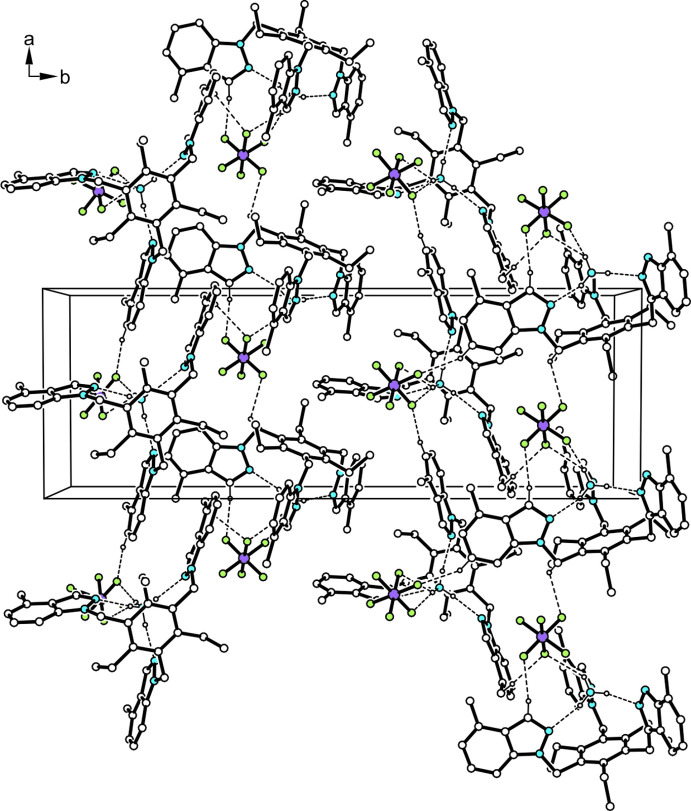
Packing excerpt of the title complex viewed down the crystallographic *c*-axis. For the sake of clarity, hydrogen atoms of the receptor not involved in hydrogen bonding are omitted.

The crystal is composed of one-dimensional supra­molecular aggregates of C—H⋯F-bonded ammonium complexes [*d*(H⋯F) 2.33–2.52 Å; Table 1 ▸] extending in the *a*-axis direction. The packing is shown in Fig. 3 ▸. Multiple π–π arene contacts connect these aggregates into a three-dimensional network. For the analysis of this type of inter­actions, the *PLATON* program (Spek, 2020 ▸) was used. The centroid–centroid distances between the inter­acting indazole units range from 3.776 (4) to 4.257 (4) Å with shifts of 1.154–1.830 Å.

## 4. Database survey

A search in the Cambridge Structural Database (CSD, Version 5.40, updated February 2019; Groom *et al.*, 2016 ▸) for ammonium complexes of 1,3,5-substituted 2,4,6-tri­alkyl­benzenes bearing pyrazolyl or indazolyl units gave eight hits, all of which contain complexes with NH_4_PF_6_. The complexes of 1,3,5-tris­[(3,5-dimethyl-1*H*-pyrazol-1-yl)meth­yl]-2,4,6-tri­ethyl­benz­ene (CUKTUX; Chin *et al.*, 1999 ▸), 1,3,5-tris­[(4-bromo-3,5-di­methyl-1*H*-pyrazol-1-yl)meth­yl]-2,4,6-tri­ethyl­benzene (UFO­HOM; Chin *et al.*, 2002 ▸), 1,3,5-tris­[(1*H*-pyrazol-1-yl)meth­yl]-2,4,6-tri­methyl­benzene (QIFFAP; Schulze *et al.*, 2018 ▸), 1,3,5-tris­[(3,5-dimethyl-1*H*-pyrazol-1-yl)meth­yl]-2,4,6-tri­methyl­ben­z­ene (QIDTOP; Schulze *et al.*, 2018 ▸) and 1,3,5-tris­[(4-bromo-3,5-dimethyl-1*H*-pyrazol-1-yl)meth­yl]-2,4,6-tri­methyl­benzene (QIDTUV; Schulze *et al.*, 2018 ▸) have the most similar structures to that of the title complex. In the crystal structure of the complex formed by 1,3,5-tris­[(4-bromo-3,5-dimethyl-1*H*-pyrazol-1-yl)meth­yl]-2,4,6-tri­ethyl­benzene (QIDVOR; Schulze *et al.*, 2018 ▸), which also contains aceto­nitrile mol­ecules, the complex lacks NH_4_^+^⋯F_6_P^−^ inter­actions. Instead, one of the solvent mol­ecules is connected to the cation *via* N—H⋯N bonding whereas the PF_6_^−^ ion is coordinated to the receptor and the solvent mol­ecules. In the crystal of the complex of 1,3,5-tris­[(3,5-diphenyl-1*H*-pyrazol-1-yl)meth­yl]-2,4,6-tri­ethyl­benzene (XEKBEX; Jonah *et al.*, 2017 ▸) the steric demand of the phenyl groups attached to the pyrazole rings prevents cation–anion inter­actions. In the case of the complex of 1,3,5-tris­[(1*H*-indazol-1-yl)meth­yl]-2,4,6-tri­ethyl­benzene (QIDVAD; Schulze *et al.*, 2018 ▸), the asymmetric unit contains four receptor mol­ecules, four NH_4_PF_6_, one water and two methanol mol­ecules. As a result, complexes are formed, the structures of which differ strongly from that of the title complex.

## 5. Synthesis and crystallization

To a solution of 4-methyl-1*H*-indazole (500 mg, 3.78 mmol) in di­methyl­formamide (9.0 mL) sodium hydroxide (152 mg, 3.78 mmol) was added and the suspension was stirred at room temperature for 30 minutes. Then 1,3,5-tris­(bromo­meth­yl)-2,4,6-tri­ethyl­benzene (418 mg, 0.95 mmol) was added and the mixture stirred at 343 K for 24 h. After cooling to room temperature, the reaction mixture was poured into ice–water (55 mL). The precipitate was filtered off, washed with a little ice–water and dried under reduced pressure. The crude mixture, containing the desired product as well as two other tri­ethyl­benzene derivatives and the unreacted 4-methyl-1*H*-indazole, was separated chromatographically. The first flash chromatography (SiO_2_; gradient, hexa­ne/ethyl acetate 4:1 to 3:2, *v*/*v*) allowed the isolation of the by-products 2,4,6-tri­ethyl-1,3-bis­[(4-methyl-1*H*-indazol-1-yl)meth­yl]-5[(4-methyl-2*H*-indazol-2-yl)meth­yl]­benzene and 2,4,6-tri­ethyl-1,3-bis­[(4-methyl-2*H*-indazol-2-yl)meth­yl]-5[(4-methyl-1*H*-indazol-1-yl]meth­yl]­benzene, while the second one (SiO_2_; gradient, toluene/ethyl acetate 16:1 to 4:1, *v*/*v*) enabled the removal of 4-methyl-1*H*-indazole. After crystallization from hexa­ne/ethyl acetate (2:1, *v*/*v*) the title compound was obtained as colourless crystals (110 mg, 20%); m.p. 427–429 K. ^1^H NMR (500 MHz, CDCl_3_, ppm) *δ* = 0.80 (*t*, *J* = 7.5 Hz, 9H), 2.58 (*s*, 9H), 2.89 (*q*, *J* = 7.5 Hz, 6H), 5.63 (*s*, 6H), 6.87 (*dt*, *J* = 7.0/1.0 Hz, 3H), 7.03 (*dd*, *J* = 8.5/1.0 Hz, 3H), 7.12 (*dd*, *J* = 8.5/7.0 Hz, 3H), 8.01 (*d*, *J* = 1.0 Hz, 3H). ^13^C NMR (125 MHz, CDCl_3_, ppm) *δ* = 14.7, 18.6, 23.8, 47.6, 107.0, 120.5, 124.8, 126.5, 130.6, 131.4, 132.0, 139.6, 145.7.

## 6. Refinement

Crystal data, data collection and structure refinement details are summarized in Table 2 ▸. The hydrogen atoms were positioned geometrically and refined isotropically using the riding model with C—H = 0.95–0.99 Å and *U*_iso_(H) = 1.2 or 1.5*U*_eq_(C). The hydrogen atoms of the ammonium ions were located in a difference-Fourier map and the N—H bond lengths refined to a target value of 0.90 Å. The crystal studied was refined as a two-component twin.

## Supplementary Material

Crystal structure: contains datablock(s) I. DOI: 10.1107/S2056989022006867/ex2059sup1.cif

Structure factors: contains datablock(s) I. DOI: 10.1107/S2056989022006867/ex2059Isup2.hkl

Supporting information file. DOI: 10.1107/S2056989022006867/ex2059Isup3.cml

CCDC reference: 2184249

Additional supporting information:  crystallographic information; 3D view; checkCIF report

## Figures and Tables

**Table 1:** Hydrogen-bond geometry (Å, °) *Cg*1, *Cg*2 and *Cg*3 represent the centroids of the N3/N4/C18/C19/C24, N3*A*/N4*A*/C18*A*/C19*A*/C24*A* and N5*A*/N6*A*/C28*A*/C29*A*/C34*A* rings, respectively.

*D*—H⋯*A*	*D*—H	H⋯*A*	*D*⋯*A*	*D*—H⋯*A*
N7—H7*A*⋯N2^i^	0.91 (2)	2.02 (3)	2.923 (8)	171 (9)
N7—H7*B*⋯N4^i^	0.91 (2)	2.10 (4)	2.980 (8)	162 (8)
N7—H7*C*⋯N6^i^	0.91 (2)	2.01 (2)	2.923 (9)	175 (9)
N7—H7*D*⋯F3	0.91 (2)	2.06 (3)	2.951 (8)	164 (7)
N7—H7*D*⋯F5	0.91 (2)	2.54 (6)	3.213 (8)	131 (6)
C13—H13⋯F6*A*	0.95	2.55	3.500 (9)	176
C17—H17*A*⋯F1	0.99	2.52	3.156 (7)	122
C17—H17*B*⋯F6	0.99	2.60	3.179 (8)	117
C18—H18⋯F2^ii^	0.95	2.33	3.201 (8)	151
C22—H22⋯F5*A* ^iii^	0.95	2.45	3.325 (8)	154
C23—H23⋯F4	0.95	2.60	3.552 (8)	175
C31—H31⋯F3^iv^	0.95	2.35	3.289 (8)	173
C37—H37*C*⋯F2*A* ^ii^	0.98	2.51	3.379 (9)	148
N7*A*—H7*AA*⋯N2*A* ^v^	0.91 (2)	2.02 (3)	2.917 (8)	171 (10)
N7*A*—H7*AB*⋯N4*A* ^v^	0.91 (2)	2.09 (3)	2.969 (8)	161 (8)
N7*A*—H7*AC*⋯N6*A* ^v^	0.92 (2)	2.03 (3)	2.941 (7)	172 (8)
N7*A*—H7*AD*⋯F3*A*	0.91 (2)	2.17 (3)	3.050 (7)	164 (6)
N7*A*—H7*AD*⋯F2*A*	0.91 (2)	2.43 (6)	3.096 (7)	130 (6)
C17*A*—H17*D*⋯F1*A*	0.99	2.59	3.247 (7)	124
C18*A*—H18*A*⋯F6*A* ^vi^	0.95	2.44	3.317 (8)	154
C23*A*—H23*A*⋯F4*A*	0.95	2.61	3.561 (8)	177
C36—H36*B*⋯*Cg*2	0.98	2.77	3.481 (7)	130
C26*A*—H26*E*⋯*Cg*3	0.98	2.66	3.592 (7)	158
C26*A*—H26*F*⋯*Cg*2	0.98	2.56	3.506 (7)	163
C36*A*—H36*F*⋯*Cg*1^vii^	0.98	2.82	3.605 (7)	138

Symmetry codes: (i) 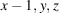 ; (ii) 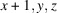 ; (iii) 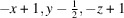 ; (iv) 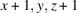 ; (v) 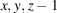 ; (vi) 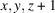 ; (vii) 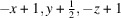 .

**Table 2:** Experimental details

Crystal data	
Chemical formula	C_39_H_42_N_6_·NH_4_^+^·PF_6_^−^
*M*_r_	757.80
Crystal system, space group	Monoclinic, *P*2_1_
Temperature (K)	123
*a*, *b*, *c* (Å)	11.1251 (11), 31.590 (2), 11.1558 (11)
β (°)	92.751 (8)
*V* (Å^3^)	3916.1 (6)
*Z*	4
Radiation type	Mo *K*α
μ (mm^−1^)	0.14
Crystal size (mm)	0.28 × 0.25 × 0.18

Data collection	
Diffractometer	Stoe *IPDS* 2T
Absorption correction	–
No. of measured, independent and observed [*I* > 2σ(*I*)] reflections	30357, 7337, 22778
*R*_int_	0.035
(sin θ/λ)_max_ (Å^−1^)	0.606

Refinement	
*R*[*F* ^2^ > 2σ(*F* ^2^)], *wR*(*F* ^2^), *S*	0.048, 0.118, 1.06
No. of reflections	30357
No. of parameters	1000
No. of restraints	9
H-atom treatment	H atoms treated by a mixture of independent and constrained refinement
Δρ_max_, Δρ_min_ (e Å^−3^)	0.35, −0.47
Absolute structure	Flack *x* determined using 3722 quotients [(*I* ^+^)−(*I* ^−^)]/[(*I* ^+^)+(*I* ^−^)] (Parsons *et al.*, 2013 ▸)
Absolute structure parameter	−0.13 (5)

Computer programs: *X-AREA* and *X-RED* (Stoe & Cie, 2002 ▸), *SHELXT2018/3* (Sheldrick, 2015*a* ▸), *SHELXL2018/3* (Sheldrick, 2015*b* ▸) and *ORTEP-3 for Windows* (Farrugia, 2012 ▸).

## supplementary crystallographic information

### Crystal data

**Table:**

C_39_H_42_N_6_·NH_4_^+^·PF_6_^−^	*F*(000) = 1592
*M**_r_* = 757.80	*D*_x_ = 1.285 Mg m^−^^3^
Monoclinic, *P*2_1_	Mo *K*α radiation, λ = 0.71073 Å
*a* = 11.1251 (11) Å	Cell parameters from 19655 reflections
*b* = 31.590 (2) Å	θ = 2.0–22.5°
*c* = 11.1558 (11) Å	µ = 0.14 mm^−^^1^
β = 92.751 (8)°	*T* = 123 K
*V* = 3916.1 (6) Å^3^	Irregular, colorless
*Z* = 4	0.28 × 0.25 × 0.18 mm

### Data collection

**Table:**

Stoe IPDS 2T diffractometer	22778 reflections with *I* > 2σ(*I*)
Radiation source: sealed X-ray tube, 12 x 0.4 mm long-fine focus	*R*_int_ = 0.035
Plane graphite monochromator	θ_max_ = 25.5°, θ_min_ = 2.6°
Detector resolution: 6.67 pixels mm^-1^	*h* = −13→13
rotation method scans	*k* = −34→38
30357 measured reflections	*l* = −13→13
7337 independent reflections	

### Refinement

**Table:**

Refinement on *F*^2^	Hydrogen site location: mixed
Least-squares matrix: full	H atoms treated by a mixture of independent and constrained refinement
*R*[*F*^2^ > 2σ(*F*^2^)] = 0.048	*w* = 1/[σ^2^(*F*_o_^2^) + (0.0264*P*)^2^ + 2.6942*P*] where *P* = (*F*_o_^2^ + 2*F*_c_^2^)/3
*wR*(*F*^2^) = 0.118	(Δ/σ)_max_ < 0.001
*S* = 1.06	Δρ_max_ = 0.35 e Å^−^^3^
30357 reflections	Δρ_min_ = −0.47 e Å^−^^3^
1000 parameters	Absolute structure: Flack *x* determined using 3722 quotients [(*I*^+^)-(*I*^-^)]/[(*I*^+^)+(*I*^-^)] (Parsons *et al.*, 2013)
9 restraints	Absolute structure parameter: −0.13 (5)

### Special details

**Table:**

Geometry. All esds (except the esd in the dihedral angle between two l.s. planes) are estimated using the full covariance matrix. The cell esds are taken into account individually in the estimation of esds in distances, angles and torsion angles; correlations between esds in cell parameters are only used when they are defined by crystal symmetry. An approximate (isotropic) treatment of cell esds is used for estimating esds involving l.s. planes.
Refinement. Refined as a 2-component twin.

### Fractional atomic coordinates and isotropic or equivalent isotropic displacement parameters (Å^2^)

**Table:**

	*x*	*y*	*z*	*U*_iso_*/*U*_eq_	
N1	0.9085 (4)	0.49651 (17)	0.3802 (5)	0.0304 (12)	
N2	1.0161 (4)	0.48419 (18)	0.4341 (5)	0.0348 (13)	
N3	0.7756 (4)	0.32258 (16)	0.5118 (4)	0.0266 (11)	
N4	0.8876 (4)	0.33661 (17)	0.4826 (5)	0.0296 (12)	
N5	0.9558 (5)	0.42548 (19)	0.9122 (5)	0.0392 (14)	
N6	1.0494 (5)	0.4218 (2)	0.8367 (6)	0.0471 (16)	
C1	0.7875 (5)	0.46663 (19)	0.5401 (5)	0.0266 (13)	
C2	0.7354 (5)	0.42832 (19)	0.4989 (5)	0.0265 (13)	
C3	0.7276 (5)	0.3944 (2)	0.5786 (5)	0.0268 (13)	
C4	0.7670 (5)	0.3987 (2)	0.7000 (5)	0.0275 (13)	
C5	0.8109 (5)	0.4377 (2)	0.7412 (5)	0.0291 (14)	
C6	0.8262 (5)	0.4718 (2)	0.6611 (6)	0.0290 (14)	
C7	0.8034 (5)	0.5025 (2)	0.4525 (6)	0.0298 (14)	
H7E	0.7304	0.5047	0.3985	0.036*	
H7F	0.8122	0.5294	0.4975	0.036*	
C8	1.0981 (6)	0.4895 (2)	0.3517 (6)	0.0357 (16)	
H8	1.1813	0.4833	0.3646	0.043*	
C9	1.0454 (6)	0.5055 (2)	0.2423 (6)	0.0321 (15)	
C10	1.0879 (6)	0.5153 (2)	0.1284 (6)	0.0385 (16)	
C11	1.0028 (7)	0.5307 (2)	0.0453 (6)	0.0434 (18)	
H11	1.0279	0.5381	−0.0321	0.052*	
C12	0.8803 (7)	0.5360 (2)	0.0698 (6)	0.0402 (17)	
H12	0.8261	0.5468	0.0090	0.048*	
C13	0.8378 (6)	0.5256 (2)	0.1802 (6)	0.0351 (15)	
H13	0.7555	0.5289	0.1972	0.042*	
C14	0.9221 (6)	0.5102 (2)	0.2652 (5)	0.0300 (14)	
C15	0.6879 (5)	0.4241 (2)	0.3692 (5)	0.0339 (15)	
H15A	0.7032	0.3951	0.3405	0.041*	
H15B	0.7316	0.4441	0.3185	0.041*	
C16	0.5531 (6)	0.4335 (3)	0.3563 (6)	0.0441 (18)	
H16A	0.5265	0.4323	0.2713	0.066*	
H16B	0.5373	0.4617	0.3882	0.066*	
H16C	0.5090	0.4123	0.4010	0.066*	
C17	0.6785 (5)	0.35214 (19)	0.5355 (6)	0.0297 (14)	
H17A	0.6274	0.3400	0.5971	0.036*	
H17B	0.6276	0.3563	0.4612	0.036*	
C18	0.9527 (5)	0.3017 (2)	0.4662 (5)	0.0315 (14)	
H18	1.0343	0.3017	0.4446	0.038*	
C19	0.8845 (5)	0.2646 (2)	0.4851 (5)	0.0273 (13)	
C20	0.9092 (5)	0.2207 (2)	0.4841 (5)	0.0282 (14)	
C21	0.8169 (5)	0.1942 (2)	0.5128 (6)	0.0341 (15)	
H21	0.8305	0.1645	0.5138	0.041*	
C22	0.7016 (6)	0.2098 (2)	0.5411 (6)	0.0342 (15)	
H22	0.6405	0.1901	0.5596	0.041*	
C23	0.6761 (5)	0.2522 (2)	0.5422 (6)	0.0314 (14)	
H23	0.5991	0.2626	0.5609	0.038*	
C24	0.7701 (5)	0.2794 (2)	0.5144 (5)	0.0263 (13)	
C25	0.7677 (6)	0.3604 (2)	0.7833 (6)	0.0351 (15)	
H25A	0.8347	0.3638	0.8442	0.042*	
H25B	0.7846	0.3349	0.7356	0.042*	
C26	0.6512 (6)	0.3529 (3)	0.8488 (6)	0.0439 (18)	
H26A	0.5832	0.3508	0.7899	0.066*	
H26B	0.6380	0.3765	0.9035	0.066*	
H26C	0.6582	0.3265	0.8947	0.066*	
C27	0.8407 (6)	0.4431 (2)	0.8731 (6)	0.0383 (16)	
H27A	0.8403	0.4737	0.8926	0.046*	
H27B	0.7770	0.4294	0.9186	0.046*	
C28	1.1413 (6)	0.4071 (3)	0.9048 (8)	0.052 (2)	
H28	1.2185	0.4010	0.8765	0.062*	
C29	1.1087 (7)	0.4017 (2)	1.0265 (7)	0.0446 (18)	
C30	1.1714 (7)	0.3876 (2)	1.1362 (8)	0.051 (2)	
C31	1.1012 (7)	0.3872 (3)	1.2350 (7)	0.0505 (19)	
H31	1.1390	0.3792	1.3098	0.061*	
C32	0.9806 (8)	0.3976 (3)	1.2336 (7)	0.056 (2)	
H32	0.9379	0.3948	1.3049	0.067*	
C33	0.9214 (7)	0.4118 (2)	1.1306 (6)	0.0485 (19)	
H33	0.8392	0.4200	1.1290	0.058*	
C34	0.9888 (7)	0.4136 (2)	1.0285 (6)	0.0396 (17)	
C35	0.8893 (6)	0.5120 (2)	0.7032 (6)	0.0344 (15)	
H35A	0.9403	0.5223	0.6389	0.041*	
H35B	0.9429	0.5054	0.7742	0.041*	
C36	0.8030 (6)	0.5476 (2)	0.7363 (6)	0.0415 (17)	
H36A	0.8495	0.5726	0.7624	0.062*	
H36B	0.7536	0.5380	0.8015	0.062*	
H36C	0.7508	0.5548	0.6661	0.062*	
C37	1.2173 (6)	0.5090 (3)	0.1021 (7)	0.049 (2)	
H37A	1.2309	0.5185	0.0202	0.074*	
H37B	1.2376	0.4789	0.1095	0.074*	
H37C	1.2683	0.5253	0.1592	0.074*	
C38	1.0334 (6)	0.2056 (2)	0.4556 (6)	0.0400 (17)	
H38A	1.0337	0.1746	0.4501	0.060*	
H38B	1.0915	0.2147	0.5192	0.060*	
H38C	1.0557	0.2177	0.3788	0.060*	
C39	1.2989 (7)	0.3748 (3)	1.1338 (9)	0.069 (3)	
H39A	1.3078	0.3531	1.0721	0.104*	
H39B	1.3255	0.3634	1.2123	0.104*	
H39C	1.3481	0.3995	1.1153	0.104*	
P1	0.32584 (14)	0.32786 (6)	0.55803 (18)	0.0391 (4)	
F1	0.4117 (4)	0.36199 (16)	0.6156 (5)	0.0707 (15)	
F2	0.2398 (4)	0.2950 (3)	0.4939 (9)	0.160 (4)	
F3	0.2545 (5)	0.3639 (2)	0.4858 (4)	0.102 (2)	
F4	0.3958 (5)	0.2928 (2)	0.6297 (6)	0.105 (2)	
F5	0.2338 (4)	0.33518 (17)	0.6590 (5)	0.0798 (17)	
F6	0.4149 (4)	0.32195 (18)	0.4524 (4)	0.0685 (15)	
N7	0.0426 (5)	0.4071 (2)	0.5778 (6)	0.0366 (13)	
H7A	0.039 (8)	0.4325 (17)	0.539 (8)	0.10 (4)*	
H7B	−0.018 (6)	0.389 (2)	0.554 (8)	0.08 (3)*	
H7C	0.041 (7)	0.413 (3)	0.658 (3)	0.08 (3)*	
H7D	0.116 (4)	0.396 (2)	0.562 (7)	0.06 (2)*	
N1A	0.6736 (4)	0.74922 (16)	0.8989 (4)	0.0280 (11)	
N2A	0.6283 (4)	0.73648 (18)	1.0056 (5)	0.0324 (12)	
N3A	0.4984 (4)	0.57729 (16)	0.7628 (4)	0.0271 (11)	
N4A	0.5334 (4)	0.59050 (16)	0.8767 (4)	0.0299 (12)	
N5A	0.1345 (4)	0.68517 (17)	0.9421 (4)	0.0284 (12)	
N6A	0.2162 (4)	0.68319 (18)	1.0390 (5)	0.0334 (13)	
C1A	0.4987 (5)	0.72111 (19)	0.7775 (5)	0.0251 (13)	
C2A	0.5286 (5)	0.68253 (19)	0.7258 (5)	0.0255 (13)	
C3A	0.4406 (5)	0.65035 (19)	0.7139 (5)	0.0250 (13)	
C4A	0.3227 (5)	0.6572 (2)	0.7494 (5)	0.0269 (13)	
C5A	0.2945 (5)	0.6967 (2)	0.7980 (5)	0.0264 (13)	
C6A	0.3817 (5)	0.72862 (19)	0.8166 (5)	0.0263 (13)	
C7A	0.5933 (5)	0.7559 (2)	0.7927 (5)	0.0294 (14)	
H7AE	0.6417	0.7568	0.7205	0.035*	
H7AF	0.5525	0.7836	0.7996	0.035*	
C8A	0.7164 (5)	0.7422 (2)	1.0901 (6)	0.0326 (15)	
H8A	0.7096	0.7357	1.1727	0.039*	
C9A	0.8212 (5)	0.7593 (2)	1.0407 (6)	0.0296 (14)	
C10A	0.9367 (5)	0.7707 (2)	1.0880 (6)	0.0331 (15)	
C11A	1.0136 (6)	0.7872 (2)	1.0064 (6)	0.0372 (16)	
H11A	1.0926	0.7950	1.0340	0.045*	
C12A	0.9802 (5)	0.7930 (2)	0.8837 (6)	0.0364 (16)	
H12A	1.0360	0.8054	0.8323	0.044*	
C13A	0.8678 (5)	0.7810 (2)	0.8369 (6)	0.0333 (15)	
H13A	0.8457	0.7843	0.7541	0.040*	
C14A	0.7891 (5)	0.7640 (2)	0.9170 (5)	0.0269 (13)	
C15A	0.6532 (5)	0.6762 (2)	0.6767 (6)	0.0338 (15)	
H15C	0.6806	0.6468	0.6932	0.041*	
H15D	0.7111	0.6958	0.7178	0.041*	
C16A	0.6503 (6)	0.6844 (3)	0.5414 (6)	0.0411 (17)	
H16D	0.7284	0.6769	0.5102	0.062*	
H16E	0.6335	0.7144	0.5258	0.062*	
H16F	0.5872	0.6670	0.5016	0.062*	
C17A	0.4759 (6)	0.6079 (2)	0.6649 (5)	0.0311 (14)	
H17C	0.4106	0.5972	0.6094	0.037*	
H17D	0.5494	0.6111	0.6191	0.037*	
C18A	0.5432 (5)	0.5556 (2)	0.9440 (6)	0.0329 (15)	
H18A	0.5667	0.5552	1.0270	0.040*	
C19A	0.5136 (5)	0.5190 (2)	0.8743 (6)	0.0298 (14)	
C20A	0.5069 (6)	0.4752 (2)	0.8989 (6)	0.0347 (15)	
C21A	0.4720 (6)	0.4496 (2)	0.8037 (6)	0.0426 (17)	
H21A	0.4657	0.4199	0.8170	0.051*	
C22A	0.4449 (6)	0.4654 (2)	0.6864 (6)	0.0407 (17)	
H22A	0.4213	0.4462	0.6241	0.049*	
C23A	0.4521 (6)	0.5079 (2)	0.6607 (6)	0.0365 (16)	
H23A	0.4353	0.5187	0.5821	0.044*	
C24A	0.4857 (5)	0.5345 (2)	0.7572 (5)	0.0275 (13)	
C25A	0.2276 (6)	0.6230 (2)	0.7317 (6)	0.0366 (16)	
H25C	0.2454	0.6064	0.6594	0.044*	
H25D	0.1485	0.6369	0.7160	0.044*	
C26A	0.2175 (6)	0.5923 (2)	0.8377 (6)	0.0375 (16)	
H26D	0.1556	0.5711	0.8177	0.056*	
H26E	0.1956	0.6082	0.9090	0.056*	
H26F	0.2950	0.5782	0.8539	0.056*	
C27A	0.1663 (5)	0.7057 (2)	0.8315 (6)	0.0342 (15)	
H27C	0.1557	0.7366	0.8401	0.041*	
H27D	0.1103	0.6960	0.7655	0.041*	
C28A	0.1567 (5)	0.6664 (2)	1.1291 (6)	0.0334 (15)	
H28A	0.1915	0.6607	1.2069	0.040*	
C29A	0.0342 (5)	0.6583 (2)	1.0929 (5)	0.0270 (13)	
C30A	−0.0658 (5)	0.6416 (2)	1.1511 (6)	0.0313 (14)	
C31A	−0.1721 (6)	0.6383 (2)	1.0830 (6)	0.0344 (15)	
H31A	−0.2416	0.6279	1.1195	0.041*	
C32A	−0.1808 (5)	0.6499 (2)	0.9607 (6)	0.0350 (15)	
H32A	−0.2555	0.6463	0.9169	0.042*	
C33A	−0.0843 (5)	0.6662 (2)	0.9025 (6)	0.0314 (14)	
H33A	−0.0905	0.6740	0.8202	0.038*	
C34A	0.0241 (5)	0.67062 (19)	0.9726 (5)	0.0265 (14)	
C35A	0.3533 (6)	0.7695 (2)	0.8800 (6)	0.0323 (14)	
H35C	0.4262	0.7792	0.9266	0.039*	
H35D	0.2900	0.7640	0.9375	0.039*	
C36A	0.3105 (6)	0.8051 (2)	0.7941 (6)	0.0374 (16)	
H36D	0.3742	0.8118	0.7395	0.056*	
H36E	0.2917	0.8304	0.8406	0.056*	
H36F	0.2383	0.7959	0.7475	0.056*	
C37A	0.9719 (6)	0.7650 (2)	1.2184 (6)	0.0438 (18)	
H37D	0.9614	0.7353	1.2407	0.066*	
H37E	0.9210	0.7829	1.2667	0.066*	
H37F	1.0564	0.7731	1.2329	0.066*	
C38A	0.5366 (7)	0.4596 (2)	1.0237 (6)	0.0450 (18)	
H38D	0.5299	0.4287	1.0256	0.067*	
H38E	0.4804	0.4720	1.0789	0.067*	
H38F	0.6190	0.4680	1.0482	0.067*	
C39A	−0.0542 (6)	0.6280 (2)	1.2795 (6)	0.0419 (17)	
H39D	−0.0008	0.6034	1.2870	0.063*	
H39E	−0.1337	0.6205	1.3072	0.063*	
H39F	−0.0204	0.6512	1.3286	0.063*	
P1A	0.48458 (15)	0.58198 (6)	0.30911 (15)	0.0329 (4)	
F1A	0.5815 (3)	0.57382 (14)	0.4164 (3)	0.0486 (11)	
F2A	0.3906 (3)	0.59112 (13)	0.1991 (3)	0.0440 (10)	
F3A	0.5780 (4)	0.61227 (17)	0.2465 (4)	0.0660 (14)	
F4A	0.3912 (4)	0.55177 (17)	0.3700 (4)	0.0655 (13)	
F5A	0.4331 (4)	0.62081 (14)	0.3796 (4)	0.0568 (12)	
F6A	0.5353 (5)	0.54260 (15)	0.2386 (4)	0.0665 (14)	
N7A	0.4738 (5)	0.66284 (19)	0.0331 (5)	0.0311 (12)	
H7AA	0.517 (8)	0.686 (2)	0.017 (10)	0.11 (4)*	
H7AB	0.488 (7)	0.645 (2)	−0.029 (5)	0.07 (3)*	
H7AC	0.392 (2)	0.668 (3)	0.028 (8)	0.07 (3)*	
H7AD	0.491 (6)	0.650 (2)	0.104 (4)	0.05 (2)*	

### Atomic displacement parameters (Å^2^)

**Table:**

	*U*^11^	*U*^22^	*U*^33^	*U*^12^	*U*^13^	*U*^23^
N1	0.034 (3)	0.032 (3)	0.025 (3)	0.002 (2)	0.003 (2)	0.006 (2)
N2	0.032 (3)	0.039 (3)	0.033 (3)	0.005 (2)	0.001 (2)	0.002 (3)
N3	0.022 (3)	0.025 (3)	0.033 (3)	0.002 (2)	0.0044 (19)	−0.002 (2)
N4	0.024 (3)	0.033 (3)	0.032 (3)	0.001 (2)	0.004 (2)	0.000 (2)
N5	0.049 (4)	0.043 (4)	0.025 (3)	−0.005 (3)	−0.004 (2)	0.002 (3)
N6	0.038 (3)	0.053 (4)	0.049 (4)	−0.004 (3)	−0.011 (3)	0.011 (3)
C1	0.028 (3)	0.025 (3)	0.027 (3)	0.003 (2)	0.003 (2)	0.004 (3)
C2	0.029 (3)	0.024 (3)	0.026 (3)	0.005 (2)	0.003 (2)	0.004 (3)
C3	0.025 (3)	0.030 (4)	0.027 (3)	0.004 (2)	0.004 (2)	0.001 (3)
C4	0.032 (3)	0.028 (3)	0.024 (3)	0.004 (2)	0.004 (2)	0.003 (3)
C5	0.035 (4)	0.027 (4)	0.024 (3)	0.001 (3)	0.001 (2)	0.003 (3)
C6	0.032 (3)	0.025 (3)	0.030 (4)	0.003 (3)	0.003 (2)	0.000 (3)
C7	0.031 (3)	0.031 (4)	0.029 (4)	0.002 (3)	0.009 (3)	0.003 (3)
C8	0.035 (4)	0.038 (4)	0.034 (4)	−0.003 (3)	0.006 (3)	−0.001 (3)
C9	0.040 (4)	0.029 (4)	0.028 (4)	−0.007 (3)	0.009 (3)	0.000 (3)
C10	0.046 (4)	0.036 (4)	0.035 (4)	−0.016 (3)	0.013 (3)	−0.004 (3)
C11	0.066 (5)	0.039 (4)	0.026 (4)	−0.017 (3)	0.007 (3)	0.001 (3)
C12	0.063 (5)	0.030 (4)	0.028 (4)	−0.006 (3)	0.000 (3)	0.002 (3)
C13	0.046 (4)	0.028 (4)	0.031 (4)	−0.006 (3)	−0.002 (3)	0.003 (3)
C14	0.042 (4)	0.025 (3)	0.023 (3)	−0.002 (3)	0.005 (3)	0.001 (3)
C15	0.044 (4)	0.035 (4)	0.023 (3)	−0.001 (3)	−0.001 (3)	0.003 (3)
C16	0.048 (4)	0.052 (5)	0.031 (4)	0.001 (3)	−0.010 (3)	0.006 (3)
C17	0.027 (3)	0.027 (3)	0.035 (4)	0.004 (2)	0.003 (2)	0.000 (3)
C18	0.031 (3)	0.033 (4)	0.031 (3)	0.002 (3)	0.003 (2)	0.001 (3)
C19	0.025 (3)	0.030 (4)	0.027 (3)	0.000 (2)	0.001 (2)	−0.002 (3)
C20	0.028 (3)	0.033 (4)	0.024 (3)	0.006 (3)	−0.003 (2)	−0.003 (3)
C21	0.036 (4)	0.030 (4)	0.035 (4)	0.001 (3)	−0.004 (3)	−0.003 (3)
C22	0.035 (4)	0.028 (4)	0.039 (4)	−0.005 (3)	−0.001 (3)	0.002 (3)
C23	0.026 (3)	0.034 (4)	0.035 (4)	−0.004 (3)	0.002 (2)	0.003 (3)
C24	0.024 (3)	0.027 (3)	0.027 (3)	−0.002 (2)	0.000 (2)	0.000 (3)
C25	0.041 (4)	0.031 (4)	0.033 (4)	0.004 (3)	0.000 (3)	0.003 (3)
C26	0.045 (4)	0.049 (5)	0.039 (4)	0.001 (3)	0.009 (3)	0.013 (3)
C27	0.051 (4)	0.042 (4)	0.022 (3)	0.004 (3)	0.000 (3)	0.004 (3)
C28	0.033 (4)	0.056 (5)	0.065 (6)	−0.005 (3)	−0.012 (3)	0.012 (4)
C29	0.058 (5)	0.034 (4)	0.041 (5)	−0.013 (3)	−0.013 (3)	0.006 (3)
C30	0.062 (5)	0.025 (4)	0.064 (6)	−0.009 (3)	−0.021 (4)	0.001 (4)
C31	0.069 (5)	0.044 (5)	0.037 (5)	−0.008 (4)	−0.010 (4)	−0.001 (4)
C32	0.085 (6)	0.040 (5)	0.042 (5)	−0.004 (4)	−0.006 (4)	0.008 (4)
C33	0.072 (5)	0.041 (4)	0.032 (4)	−0.001 (4)	−0.002 (3)	−0.001 (3)
C34	0.060 (5)	0.032 (4)	0.026 (4)	−0.007 (3)	−0.010 (3)	0.000 (3)
C35	0.043 (4)	0.031 (4)	0.030 (4)	0.002 (3)	0.001 (3)	−0.002 (3)
C36	0.050 (4)	0.035 (4)	0.040 (4)	0.002 (3)	0.009 (3)	−0.006 (3)
C37	0.055 (5)	0.048 (5)	0.047 (5)	−0.015 (4)	0.025 (3)	−0.007 (4)
C38	0.038 (4)	0.038 (4)	0.044 (4)	0.009 (3)	−0.001 (3)	−0.003 (3)
C39	0.059 (6)	0.054 (6)	0.093 (7)	−0.017 (4)	−0.023 (5)	0.024 (5)
P1	0.0251 (9)	0.0406 (11)	0.0514 (12)	0.0037 (8)	−0.0008 (7)	−0.0073 (9)
F1	0.042 (2)	0.069 (3)	0.101 (4)	−0.007 (2)	0.002 (2)	−0.046 (3)
F2	0.028 (3)	0.140 (6)	0.314 (11)	−0.021 (3)	0.010 (4)	−0.149 (7)
F3	0.107 (4)	0.153 (6)	0.046 (3)	0.092 (4)	−0.006 (3)	0.009 (3)
F4	0.106 (4)	0.079 (4)	0.134 (5)	0.058 (4)	0.043 (4)	0.052 (4)
F5	0.065 (3)	0.074 (4)	0.105 (4)	0.016 (3)	0.055 (3)	0.016 (3)
F6	0.050 (3)	0.103 (4)	0.053 (3)	0.019 (3)	0.012 (2)	−0.029 (3)
N7	0.031 (3)	0.033 (4)	0.045 (4)	0.005 (3)	−0.001 (3)	0.002 (3)
N1A	0.030 (3)	0.031 (3)	0.022 (3)	−0.007 (2)	−0.001 (2)	0.003 (2)
N2A	0.032 (3)	0.039 (3)	0.026 (3)	−0.004 (2)	0.000 (2)	0.002 (2)
N3A	0.036 (3)	0.022 (3)	0.023 (3)	0.001 (2)	0.000 (2)	0.000 (2)
N4A	0.041 (3)	0.029 (3)	0.020 (3)	−0.001 (2)	0.001 (2)	0.002 (2)
N5A	0.027 (3)	0.039 (3)	0.019 (3)	−0.003 (2)	0.004 (2)	0.002 (2)
N6A	0.030 (3)	0.044 (3)	0.025 (3)	−0.003 (2)	−0.003 (2)	0.002 (3)
C1A	0.026 (3)	0.026 (3)	0.023 (3)	−0.001 (2)	−0.001 (2)	0.005 (3)
C2A	0.023 (3)	0.027 (3)	0.027 (3)	−0.001 (2)	0.001 (2)	0.000 (3)
C3A	0.031 (3)	0.021 (3)	0.023 (3)	−0.001 (2)	0.000 (2)	0.001 (3)
C4A	0.028 (3)	0.029 (4)	0.023 (3)	−0.003 (2)	−0.001 (2)	0.004 (3)
C5A	0.023 (3)	0.030 (3)	0.027 (3)	−0.001 (2)	0.000 (2)	0.003 (3)
C6A	0.026 (3)	0.026 (3)	0.026 (3)	0.003 (2)	−0.002 (2)	0.001 (3)
C7A	0.031 (3)	0.030 (4)	0.027 (3)	−0.005 (3)	−0.005 (2)	0.004 (3)
C8A	0.032 (3)	0.040 (4)	0.026 (3)	−0.002 (3)	−0.002 (3)	−0.002 (3)
C9A	0.023 (3)	0.030 (4)	0.036 (4)	0.000 (2)	0.000 (2)	−0.005 (3)
C10A	0.029 (3)	0.028 (4)	0.041 (4)	0.003 (3)	−0.007 (3)	−0.008 (3)
C11A	0.028 (3)	0.033 (4)	0.050 (4)	0.000 (3)	0.000 (3)	−0.009 (3)
C12A	0.028 (3)	0.032 (4)	0.050 (4)	−0.001 (3)	0.006 (3)	−0.002 (3)
C13A	0.027 (3)	0.030 (4)	0.043 (4)	0.000 (3)	0.003 (3)	−0.001 (3)
C14A	0.024 (3)	0.026 (3)	0.030 (3)	−0.002 (2)	−0.001 (2)	0.000 (3)
C15A	0.026 (3)	0.042 (4)	0.034 (4)	0.001 (3)	0.004 (2)	−0.007 (3)
C16A	0.036 (4)	0.057 (5)	0.032 (4)	−0.008 (3)	0.011 (3)	0.001 (3)
C17A	0.047 (4)	0.025 (3)	0.021 (3)	0.005 (3)	0.001 (3)	0.004 (3)
C18A	0.035 (4)	0.036 (4)	0.027 (4)	0.003 (3)	0.000 (3)	0.002 (3)
C19A	0.037 (4)	0.026 (3)	0.027 (3)	0.003 (3)	0.005 (2)	0.002 (3)
C20A	0.042 (4)	0.030 (4)	0.033 (4)	0.004 (3)	0.006 (3)	0.004 (3)
C21A	0.059 (5)	0.028 (4)	0.042 (4)	0.000 (3)	0.008 (3)	0.003 (3)
C22A	0.057 (5)	0.028 (4)	0.037 (4)	0.000 (3)	0.006 (3)	−0.005 (3)
C23A	0.048 (4)	0.029 (4)	0.033 (4)	−0.001 (3)	0.004 (3)	−0.007 (3)
C24A	0.034 (3)	0.023 (3)	0.026 (3)	0.002 (2)	0.002 (2)	0.000 (3)
C25A	0.041 (4)	0.036 (4)	0.033 (4)	−0.013 (3)	0.003 (3)	0.000 (3)
C26A	0.041 (4)	0.030 (4)	0.042 (4)	−0.005 (3)	0.009 (3)	0.007 (3)
C27A	0.026 (3)	0.047 (4)	0.031 (4)	−0.001 (3)	0.005 (3)	0.007 (3)
C28A	0.034 (4)	0.042 (4)	0.025 (3)	−0.003 (3)	0.004 (3)	0.001 (3)
C29A	0.026 (3)	0.028 (4)	0.027 (3)	−0.001 (2)	0.002 (2)	−0.002 (3)
C30A	0.035 (4)	0.026 (3)	0.034 (4)	−0.001 (3)	0.006 (3)	0.000 (3)
C31A	0.033 (4)	0.034 (4)	0.037 (4)	−0.003 (3)	0.007 (3)	0.002 (3)
C32A	0.028 (3)	0.036 (4)	0.041 (4)	−0.001 (3)	0.001 (3)	−0.004 (3)
C33A	0.029 (3)	0.033 (4)	0.032 (4)	0.001 (3)	0.002 (2)	−0.003 (3)
C34A	0.021 (3)	0.028 (4)	0.030 (4)	0.000 (2)	0.002 (2)	−0.004 (3)
C35A	0.041 (4)	0.028 (4)	0.028 (3)	0.002 (3)	−0.001 (3)	−0.002 (3)
C36A	0.044 (4)	0.027 (4)	0.041 (4)	0.008 (3)	0.001 (3)	0.001 (3)
C37A	0.040 (4)	0.049 (5)	0.041 (4)	0.003 (3)	−0.012 (3)	−0.015 (4)
C38A	0.067 (5)	0.031 (4)	0.038 (4)	0.004 (3)	0.009 (3)	0.007 (3)
C39A	0.047 (4)	0.044 (4)	0.035 (4)	−0.009 (3)	0.010 (3)	0.002 (3)
P1A	0.0428 (10)	0.0310 (9)	0.0249 (9)	0.0032 (7)	0.0031 (7)	0.0004 (7)
F1A	0.054 (2)	0.060 (3)	0.031 (2)	0.019 (2)	−0.0057 (16)	0.000 (2)
F2A	0.054 (2)	0.042 (3)	0.034 (2)	−0.0029 (18)	−0.0120 (17)	0.0061 (18)
F3A	0.050 (3)	0.086 (4)	0.061 (3)	−0.022 (2)	0.000 (2)	0.032 (3)
F4A	0.075 (3)	0.069 (3)	0.053 (3)	−0.024 (3)	0.007 (2)	0.024 (3)
F5A	0.070 (3)	0.046 (3)	0.054 (3)	0.026 (2)	−0.005 (2)	−0.019 (2)
F6A	0.107 (4)	0.055 (3)	0.037 (3)	0.033 (3)	0.001 (2)	−0.013 (2)
N7A	0.034 (3)	0.032 (3)	0.027 (3)	0.000 (2)	0.002 (2)	0.004 (3)

### Geometric parameters (Å, º)

**Table:**

N1—C14	1.369 (8)	N1A—C14A	1.372 (7)
N1—N2	1.370 (7)	N1A—N2A	1.376 (7)
N1—C7	1.465 (7)	N1A—C7A	1.465 (7)
N2—C8	1.336 (8)	N2A—C8A	1.339 (8)
N3—C24	1.367 (8)	N3A—C24A	1.362 (8)
N3—N4	1.377 (7)	N3A—N4A	1.377 (7)
N3—C17	1.461 (7)	N3A—C17A	1.471 (8)
N4—C18	1.337 (8)	N4A—C18A	1.335 (8)
N5—N6	1.375 (8)	N5A—C34A	1.369 (7)
N5—C34	1.383 (8)	N5A—N6A	1.380 (7)
N5—C27	1.445 (8)	N5A—C27A	1.452 (8)
N6—C28	1.327 (9)	N6A—C28A	1.339 (8)
C1—C6	1.407 (8)	C1A—C2A	1.395 (8)
C1—C2	1.410 (9)	C1A—C6A	1.413 (8)
C1—C7	1.513 (8)	C1A—C7A	1.525 (8)
C2—C3	1.398 (8)	C2A—C3A	1.413 (8)
C2—C15	1.521 (8)	C2A—C15A	1.528 (8)
C3—C4	1.409 (8)	C3A—C4A	1.405 (8)
C3—C17	1.512 (9)	C3A—C17A	1.507 (9)
C4—C5	1.396 (9)	C4A—C5A	1.403 (9)
C4—C25	1.524 (9)	C4A—C25A	1.518 (8)
C5—C6	1.414 (9)	C5A—C6A	1.407 (8)
C5—C27	1.503 (9)	C5A—C27A	1.518 (8)
C6—C35	1.516 (9)	C6A—C35A	1.514 (8)
C7—H7E	0.9900	C7A—H7AE	0.9900
C7—H7F	0.9900	C7A—H7AF	0.9900
C8—C9	1.422 (9)	C8A—C9A	1.420 (9)
C8—H8	0.9500	C8A—H8A	0.9500
C9—C10	1.412 (9)	C9A—C10A	1.411 (8)
C9—C14	1.415 (9)	C9A—C14A	1.417 (9)
C10—C11	1.380 (10)	C10A—C11A	1.382 (10)
C10—C37	1.497 (10)	C10A—C37A	1.499 (9)
C11—C12	1.412 (10)	C11A—C12A	1.413 (9)
C11—H11	0.9500	C11A—H11A	0.9500
C12—C13	1.379 (9)	C12A—C13A	1.384 (8)
C12—H12	0.9500	C12A—H12A	0.9500
C13—C14	1.390 (9)	C13A—C14A	1.390 (9)
C13—H13	0.9500	C13A—H13A	0.9500
C15—C16	1.529 (9)	C15A—C16A	1.530 (9)
C15—H15A	0.9900	C15A—H15C	0.9900
C15—H15B	0.9900	C15A—H15D	0.9900
C16—H16A	0.9800	C16A—H16D	0.9800
C16—H16B	0.9800	C16A—H16E	0.9800
C16—H16C	0.9800	C16A—H16F	0.9800
C17—H17A	0.9900	C17A—H17C	0.9900
C17—H17B	0.9900	C17A—H17D	0.9900
C18—C19	1.416 (9)	C18A—C19A	1.423 (9)
C18—H18	0.9500	C18A—H18A	0.9500
C19—C24	1.409 (8)	C19A—C20A	1.413 (9)
C19—C20	1.415 (9)	C19A—C24A	1.415 (9)
C20—C21	1.373 (9)	C20A—C21A	1.376 (10)
C20—C38	1.511 (8)	C20A—C38A	1.499 (9)
C21—C22	1.423 (9)	C21A—C22A	1.420 (10)
C21—H21	0.9500	C21A—H21A	0.9500
C22—C23	1.371 (9)	C22A—C23A	1.376 (10)
C22—H22	0.9500	C22A—H22A	0.9500
C23—C24	1.398 (8)	C23A—C24A	1.401 (9)
C23—H23	0.9500	C23A—H23A	0.9500
C25—C26	1.536 (9)	C25A—C26A	1.537 (9)
C25—H25A	0.9900	C25A—H25C	0.9900
C25—H25B	0.9900	C25A—H25D	0.9900
C26—H26A	0.9800	C26A—H26D	0.9800
C26—H26B	0.9800	C26A—H26E	0.9800
C26—H26C	0.9800	C26A—H26F	0.9800
C27—H27A	0.9900	C27A—H27C	0.9900
C27—H27B	0.9900	C27A—H27D	0.9900
C28—C29	1.432 (11)	C28A—C29A	1.426 (8)
C28—H28	0.9500	C28A—H28A	0.9500
C29—C34	1.387 (10)	C29A—C34A	1.397 (8)
C29—C30	1.449 (10)	C29A—C30A	1.417 (8)
C30—C31	1.381 (12)	C30A—C31A	1.378 (9)
C30—C39	1.477 (11)	C30A—C39A	1.496 (9)
C31—C32	1.381 (11)	C31A—C32A	1.412 (9)
C31—H31	0.9500	C31A—H31A	0.9500
C32—C33	1.372 (10)	C32A—C33A	1.381 (9)
C32—H32	0.9500	C32A—H32A	0.9500
C33—C34	1.395 (11)	C33A—C34A	1.413 (8)
C33—H33	0.9500	C33A—H33A	0.9500
C35—C36	1.534 (9)	C35A—C36A	1.538 (9)
C35—H35A	0.9900	C35A—H35C	0.9900
C35—H35B	0.9900	C35A—H35D	0.9900
C36—H36A	0.9800	C36A—H36D	0.9800
C36—H36B	0.9800	C36A—H36E	0.9800
C36—H36C	0.9800	C36A—H36F	0.9800
C37—H37A	0.9800	C37A—H37D	0.9800
C37—H37B	0.9800	C37A—H37E	0.9800
C37—H37C	0.9800	C37A—H37F	0.9800
C38—H38A	0.9800	C38A—H38D	0.9800
C38—H38B	0.9800	C38A—H38E	0.9800
C38—H38C	0.9800	C38A—H38F	0.9800
C39—H39A	0.9800	C39A—H39D	0.9800
C39—H39B	0.9800	C39A—H39E	0.9800
C39—H39C	0.9800	C39A—H39F	0.9800
P1—F4	1.552 (5)	P1A—F1A	1.593 (4)
P1—F1	1.558 (5)	P1A—F2A	1.599 (4)
P1—F2	1.561 (5)	P1A—F3A	1.598 (5)
P1—F5	1.575 (5)	P1A—F4A	1.587 (4)
P1—F3	1.585 (5)	P1A—F5A	1.579 (4)
P1—F6	1.587 (5)	P1A—F6A	1.590 (5)
N7—H7A	0.91 (2)	N7A—H7AA	0.91 (2)
N7—H7B	0.91 (2)	N7A—H7AB	0.91 (2)
N7—H7C	0.91 (2)	N7A—H7AC	0.92 (2)
N7—H7D	0.91 (2)	N7A—H7AD	0.91 (2)
			
C14—N1—N2	111.6 (5)	C14A—N1A—N2A	110.6 (4)
C14—N1—C7	127.0 (5)	C14A—N1A—C7A	126.9 (5)
N2—N1—C7	120.0 (5)	N2A—N1A—C7A	120.5 (5)
C8—N2—N1	105.8 (5)	C8A—N2A—N1A	106.4 (5)
C24—N3—N4	111.6 (5)	C24A—N3A—N4A	111.5 (5)
C24—N3—C17	126.9 (5)	C24A—N3A—C17A	127.3 (5)
N4—N3—C17	121.5 (5)	N4A—N3A—C17A	121.1 (5)
C18—N4—N3	105.6 (5)	C18A—N4A—N3A	106.3 (5)
N6—N5—C34	112.1 (6)	C34A—N5A—N6A	110.8 (5)
N6—N5—C27	122.3 (5)	C34A—N5A—C27A	128.2 (5)
C34—N5—C27	125.4 (6)	N6A—N5A—C27A	120.5 (5)
C28—N6—N5	105.3 (6)	C28A—N6A—N5A	106.0 (5)
C6—C1—C2	121.0 (5)	C2A—C1A—C6A	121.1 (5)
C6—C1—C7	119.6 (5)	C2A—C1A—C7A	119.9 (5)
C2—C1—C7	119.4 (5)	C6A—C1A—C7A	119.1 (5)
C3—C2—C1	119.1 (5)	C1A—C2A—C3A	119.3 (5)
C3—C2—C15	120.6 (6)	C1A—C2A—C15A	120.1 (5)
C1—C2—C15	120.2 (5)	C3A—C2A—C15A	120.5 (5)
C2—C3—C4	120.8 (6)	C4A—C3A—C2A	120.9 (5)
C2—C3—C17	120.4 (5)	C4A—C3A—C17A	120.2 (5)
C4—C3—C17	118.8 (5)	C2A—C3A—C17A	118.9 (5)
C5—C4—C3	119.3 (5)	C5A—C4A—C3A	118.4 (5)
C5—C4—C25	120.6 (5)	C5A—C4A—C25A	121.0 (5)
C3—C4—C25	120.1 (5)	C3A—C4A—C25A	120.5 (6)
C4—C5—C6	121.0 (6)	C4A—C5A—C6A	121.9 (5)
C4—C5—C27	118.7 (6)	C4A—C5A—C27A	119.4 (5)
C6—C5—C27	120.3 (6)	C6A—C5A—C27A	118.7 (6)
C1—C6—C5	118.5 (6)	C5A—C6A—C1A	118.3 (6)
C1—C6—C35	120.7 (6)	C5A—C6A—C35A	121.4 (5)
C5—C6—C35	120.7 (5)	C1A—C6A—C35A	120.3 (5)
N1—C7—C1	112.3 (5)	N1A—C7A—C1A	112.2 (5)
N1—C7—H7E	109.1	N1A—C7A—H7AE	109.2
C1—C7—H7E	109.1	C1A—C7A—H7AE	109.2
N1—C7—H7F	109.1	N1A—C7A—H7AF	109.2
C1—C7—H7F	109.1	C1A—C7A—H7AF	109.2
H7E—C7—H7F	107.9	H7AE—C7A—H7AF	107.9
N2—C8—C9	111.6 (6)	N2A—C8A—C9A	111.5 (6)
N2—C8—H8	124.2	N2A—C8A—H8A	124.3
C9—C8—H8	124.2	C9A—C8A—H8A	124.3
C10—C9—C14	120.6 (6)	C10A—C9A—C14A	121.3 (6)
C10—C9—C8	135.2 (6)	C10A—C9A—C8A	134.5 (6)
C14—C9—C8	104.2 (5)	C14A—C9A—C8A	104.1 (5)
C11—C10—C9	115.6 (6)	C11A—C10A—C9A	115.6 (6)
C11—C10—C37	123.4 (6)	C11A—C10A—C37A	122.9 (6)
C9—C10—C37	120.9 (6)	C9A—C10A—C37A	121.5 (6)
C10—C11—C12	123.3 (6)	C10A—C11A—C12A	123.0 (6)
C10—C11—H11	118.4	C10A—C11A—H11A	118.5
C12—C11—H11	118.4	C12A—C11A—H11A	118.5
C13—C12—C11	121.3 (6)	C13A—C12A—C11A	121.4 (6)
C13—C12—H12	119.3	C13A—C12A—H12A	119.3
C11—C12—H12	119.3	C11A—C12A—H12A	119.3
C12—C13—C14	116.3 (6)	C12A—C13A—C14A	116.7 (6)
C12—C13—H13	121.8	C12A—C13A—H13A	121.7
C14—C13—H13	121.8	C14A—C13A—H13A	121.7
N1—C14—C13	130.5 (6)	N1A—C14A—C13A	130.7 (6)
N1—C14—C9	106.8 (5)	N1A—C14A—C9A	107.4 (5)
C13—C14—C9	122.8 (6)	C13A—C14A—C9A	122.0 (5)
C2—C15—C16	111.5 (5)	C2A—C15A—C16A	110.8 (5)
C2—C15—H15A	109.3	C2A—C15A—H15C	109.5
C16—C15—H15A	109.3	C16A—C15A—H15C	109.5
C2—C15—H15B	109.3	C2A—C15A—H15D	109.5
C16—C15—H15B	109.3	C16A—C15A—H15D	109.5
H15A—C15—H15B	108.0	H15C—C15A—H15D	108.1
C15—C16—H16A	109.5	C15A—C16A—H16D	109.5
C15—C16—H16B	109.5	C15A—C16A—H16E	109.5
H16A—C16—H16B	109.5	H16D—C16A—H16E	109.5
C15—C16—H16C	109.5	C15A—C16A—H16F	109.5
H16A—C16—H16C	109.5	H16D—C16A—H16F	109.5
H16B—C16—H16C	109.5	H16E—C16A—H16F	109.5
N3—C17—C3	111.3 (5)	N3A—C17A—C3A	110.7 (5)
N3—C17—H17A	109.4	N3A—C17A—H17C	109.5
C3—C17—H17A	109.4	C3A—C17A—H17C	109.5
N3—C17—H17B	109.4	N3A—C17A—H17D	109.5
C3—C17—H17B	109.4	C3A—C17A—H17D	109.5
H17A—C17—H17B	108.0	H17C—C17A—H17D	108.1
N4—C18—C19	111.4 (5)	N4A—C18A—C19A	110.7 (5)
N4—C18—H18	124.3	N4A—C18A—H18A	124.6
C19—C18—H18	124.3	C19A—C18A—H18A	124.6
C24—C19—C20	120.3 (5)	C20A—C19A—C24A	120.4 (6)
C24—C19—C18	104.9 (5)	C20A—C19A—C18A	134.7 (6)
C20—C19—C18	134.8 (6)	C24A—C19A—C18A	104.9 (5)
C21—C20—C19	116.6 (5)	C21A—C20A—C19A	116.2 (6)
C21—C20—C38	124.0 (6)	C21A—C20A—C38A	124.5 (6)
C19—C20—C38	119.4 (6)	C19A—C20A—C38A	119.3 (6)
C20—C21—C22	122.3 (6)	C20A—C21A—C22A	122.9 (7)
C20—C21—H21	118.9	C20A—C21A—H21A	118.6
C22—C21—H21	118.9	C22A—C21A—H21A	118.6
C23—C22—C21	121.9 (6)	C23A—C22A—C21A	121.7 (7)
C23—C22—H22	119.0	C23A—C22A—H22A	119.2
C21—C22—H22	119.0	C21A—C22A—H22A	119.2
C22—C23—C24	116.1 (6)	C22A—C23A—C24A	116.0 (6)
C22—C23—H23	121.9	C22A—C23A—H23A	122.0
C24—C23—H23	121.9	C24A—C23A—H23A	122.0
N3—C24—C23	130.7 (5)	N3A—C24A—C23A	130.7 (6)
N3—C24—C19	106.5 (5)	N3A—C24A—C19A	106.5 (5)
C23—C24—C19	122.8 (6)	C23A—C24A—C19A	122.8 (6)
C4—C25—C26	115.7 (5)	C4A—C25A—C26A	115.1 (5)
C4—C25—H25A	108.4	C4A—C25A—H25C	108.5
C26—C25—H25A	108.4	C26A—C25A—H25C	108.5
C4—C25—H25B	108.4	C4A—C25A—H25D	108.5
C26—C25—H25B	108.4	C26A—C25A—H25D	108.5
H25A—C25—H25B	107.4	H25C—C25A—H25D	107.5
C25—C26—H26A	109.5	C25A—C26A—H26D	109.5
C25—C26—H26B	109.5	C25A—C26A—H26E	109.5
H26A—C26—H26B	109.5	H26D—C26A—H26E	109.5
C25—C26—H26C	109.5	C25A—C26A—H26F	109.5
H26A—C26—H26C	109.5	H26D—C26A—H26F	109.5
H26B—C26—H26C	109.5	H26E—C26A—H26F	109.5
N5—C27—C5	113.7 (5)	N5A—C27A—C5A	113.3 (5)
N5—C27—H27A	108.8	N5A—C27A—H27C	108.9
C5—C27—H27A	108.8	C5A—C27A—H27C	108.9
N5—C27—H27B	108.8	N5A—C27A—H27D	108.9
C5—C27—H27B	108.8	C5A—C27A—H27D	108.9
H27A—C27—H27B	107.7	H27C—C27A—H27D	107.7
N6—C28—C29	111.2 (7)	N6A—C28A—C29A	111.0 (5)
N6—C28—H28	124.4	N6A—C28A—H28A	124.5
C29—C28—H28	124.4	C29A—C28A—H28A	124.5
C34—C29—C28	105.7 (6)	C34A—C29A—C30A	121.0 (5)
C34—C29—C30	119.6 (7)	C34A—C29A—C28A	104.7 (5)
C28—C29—C30	134.7 (8)	C30A—C29A—C28A	134.3 (6)
C31—C30—C29	114.2 (7)	C31A—C30A—C29A	116.6 (6)
C31—C30—C39	126.3 (7)	C31A—C30A—C39A	122.6 (6)
C29—C30—C39	119.5 (8)	C29A—C30A—C39A	120.8 (5)
C32—C31—C30	125.1 (7)	C30A—C31A—C32A	122.0 (6)
C32—C31—H31	117.5	C30A—C31A—H31A	119.0
C30—C31—H31	117.5	C32A—C31A—H31A	119.0
C33—C32—C31	121.0 (8)	C33A—C32A—C31A	122.2 (6)
C33—C32—H32	119.5	C33A—C32A—H32A	118.9
C31—C32—H32	119.5	C31A—C32A—H32A	118.9
C32—C33—C34	116.3 (8)	C32A—C33A—C34A	116.0 (6)
C32—C33—H33	121.9	C32A—C33A—H33A	122.0
C34—C33—H33	121.9	C34A—C33A—H33A	122.0
N5—C34—C29	105.7 (6)	N5A—C34A—C29A	107.5 (5)
N5—C34—C33	130.4 (7)	N5A—C34A—C33A	130.4 (6)
C29—C34—C33	123.8 (6)	C29A—C34A—C33A	122.1 (5)
C6—C35—C36	113.7 (5)	C6A—C35A—C36A	113.5 (5)
C6—C35—H35A	108.8	C6A—C35A—H35C	108.9
C36—C35—H35A	108.8	C36A—C35A—H35C	108.9
C6—C35—H35B	108.8	C6A—C35A—H35D	108.9
C36—C35—H35B	108.8	C36A—C35A—H35D	108.9
H35A—C35—H35B	107.7	H35C—C35A—H35D	107.7
C35—C36—H36A	109.5	C35A—C36A—H36D	109.5
C35—C36—H36B	109.5	C35A—C36A—H36E	109.5
H36A—C36—H36B	109.5	H36D—C36A—H36E	109.5
C35—C36—H36C	109.5	C35A—C36A—H36F	109.5
H36A—C36—H36C	109.5	H36D—C36A—H36F	109.5
H36B—C36—H36C	109.5	H36E—C36A—H36F	109.5
C10—C37—H37A	109.5	C10A—C37A—H37D	109.5
C10—C37—H37B	109.5	C10A—C37A—H37E	109.5
H37A—C37—H37B	109.5	H37D—C37A—H37E	109.5
C10—C37—H37C	109.5	C10A—C37A—H37F	109.5
H37A—C37—H37C	109.5	H37D—C37A—H37F	109.5
H37B—C37—H37C	109.5	H37E—C37A—H37F	109.5
C20—C38—H38A	109.5	C20A—C38A—H38D	109.5
C20—C38—H38B	109.5	C20A—C38A—H38E	109.5
H38A—C38—H38B	109.5	H38D—C38A—H38E	109.5
C20—C38—H38C	109.5	C20A—C38A—H38F	109.5
H38A—C38—H38C	109.5	H38D—C38A—H38F	109.5
H38B—C38—H38C	109.5	H38E—C38A—H38F	109.5
C30—C39—H39A	109.5	C30A—C39A—H39D	109.5
C30—C39—H39B	109.5	C30A—C39A—H39E	109.5
H39A—C39—H39B	109.5	H39D—C39A—H39E	109.5
C30—C39—H39C	109.5	C30A—C39A—H39F	109.5
H39A—C39—H39C	109.5	H39D—C39A—H39F	109.5
H39B—C39—H39C	109.5	H39E—C39A—H39F	109.5
F4—P1—F1	89.9 (4)	F5A—P1A—F4A	89.6 (3)
F4—P1—F2	92.5 (5)	F5A—P1A—F6A	179.4 (3)
F1—P1—F2	176.9 (5)	F4A—P1A—F6A	89.8 (3)
F4—P1—F5	93.6 (3)	F5A—P1A—F1A	90.0 (2)
F1—P1—F5	90.6 (3)	F4A—P1A—F1A	90.8 (3)
F2—P1—F5	91.1 (4)	F6A—P1A—F1A	90.1 (2)
F4—P1—F3	179.5 (4)	F5A—P1A—F3A	90.7 (3)
F1—P1—F3	89.7 (4)	F4A—P1A—F3A	179.4 (3)
F2—P1—F3	87.9 (5)	F6A—P1A—F3A	89.9 (3)
F5—P1—F3	86.0 (3)	F1A—P1A—F3A	89.7 (2)
F4—P1—F6	89.0 (3)	F5A—P1A—F2A	90.1 (2)
F1—P1—F6	89.7 (3)	F4A—P1A—F2A	91.1 (2)
F2—P1—F6	88.5 (4)	F6A—P1A—F2A	89.8 (2)
F5—P1—F6	177.3 (3)	F1A—P1A—F2A	178.0 (3)
F3—P1—F6	91.3 (3)	F3A—P1A—F2A	88.4 (2)
H7C—N7—H7A	106 (9)	H7AD—N7A—H7AA	117 (8)
H7C—N7—H7D	109 (7)	H7AD—N7A—H7AB	109 (7)
H7A—N7—H7D	106 (8)	H7AA—N7A—H7AB	104 (8)
H7C—N7—H7B	111 (8)	H7AD—N7A—H7AC	108 (7)
H7A—N7—H7B	113 (8)	H7AA—N7A—H7AC	112 (9)
H7D—N7—H7B	111 (8)	H7AB—N7A—H7AC	105 (7)
			
C14—N1—N2—C8	−1.0 (7)	C14A—N1A—N2A—C8A	−1.5 (7)
C7—N1—N2—C8	−168.1 (6)	C7A—N1A—N2A—C8A	−166.5 (6)
C24—N3—N4—C18	0.3 (6)	C24A—N3A—N4A—C18A	0.1 (6)
C17—N3—N4—C18	−179.5 (5)	C17A—N3A—N4A—C18A	178.5 (5)
C34—N5—N6—C28	0.7 (8)	C34A—N5A—N6A—C28A	1.3 (7)
C27—N5—N6—C28	176.0 (6)	C27A—N5A—N6A—C28A	174.0 (6)
C6—C1—C2—C3	−3.1 (9)	C6A—C1A—C2A—C3A	−0.8 (9)
C7—C1—C2—C3	176.2 (5)	C7A—C1A—C2A—C3A	179.1 (5)
C6—C1—C2—C15	176.7 (5)	C6A—C1A—C2A—C15A	176.0 (5)
C7—C1—C2—C15	−4.0 (8)	C7A—C1A—C2A—C15A	−4.2 (8)
C1—C2—C3—C4	2.4 (8)	C1A—C2A—C3A—C4A	2.2 (9)
C15—C2—C3—C4	−177.4 (5)	C15A—C2A—C3A—C4A	−174.5 (5)
C1—C2—C3—C17	−176.6 (5)	C1A—C2A—C3A—C17A	−176.4 (5)
C15—C2—C3—C17	3.7 (8)	C15A—C2A—C3A—C17A	6.9 (8)
C2—C3—C4—C5	1.9 (9)	C2A—C3A—C4A—C5A	−0.5 (8)
C17—C3—C4—C5	−179.1 (5)	C17A—C3A—C4A—C5A	178.1 (5)
C2—C3—C4—C25	−174.9 (5)	C2A—C3A—C4A—C25A	177.6 (5)
C17—C3—C4—C25	4.1 (8)	C17A—C3A—C4A—C25A	−3.9 (8)
C3—C4—C5—C6	−5.6 (9)	C3A—C4A—C5A—C6A	−2.8 (9)
C25—C4—C5—C6	171.2 (5)	C25A—C4A—C5A—C6A	179.2 (6)
C3—C4—C5—C27	173.8 (5)	C3A—C4A—C5A—C27A	177.1 (5)
C25—C4—C5—C27	−9.4 (9)	C25A—C4A—C5A—C27A	−1.0 (9)
C2—C1—C6—C5	−0.4 (9)	C4A—C5A—C6A—C1A	4.2 (9)
C7—C1—C6—C5	−179.7 (5)	C27A—C5A—C6A—C1A	−175.7 (5)
C2—C1—C6—C35	176.1 (6)	C4A—C5A—C6A—C35A	−174.0 (6)
C7—C1—C6—C35	−3.2 (8)	C27A—C5A—C6A—C35A	6.2 (8)
C4—C5—C6—C1	4.9 (9)	C2A—C1A—C6A—C5A	−2.4 (8)
C27—C5—C6—C1	−174.6 (5)	C7A—C1A—C6A—C5A	177.8 (5)
C4—C5—C6—C35	−171.7 (6)	C2A—C1A—C6A—C35A	175.8 (5)
C27—C5—C6—C35	8.9 (9)	C7A—C1A—C6A—C35A	−4.0 (8)
C14—N1—C7—C1	149.3 (6)	C14A—N1A—C7A—C1A	154.9 (6)
N2—N1—C7—C1	−45.7 (8)	N2A—N1A—C7A—C1A	−42.8 (8)
C6—C1—C7—N1	100.0 (7)	C2A—C1A—C7A—N1A	−80.8 (7)
C2—C1—C7—N1	−79.2 (7)	C6A—C1A—C7A—N1A	99.0 (6)
N1—N2—C8—C9	0.1 (8)	N1A—N2A—C8A—C9A	0.6 (7)
N2—C8—C9—C10	−177.5 (7)	N2A—C8A—C9A—C10A	−179.2 (7)
N2—C8—C9—C14	0.7 (8)	N2A—C8A—C9A—C14A	0.6 (8)
C14—C9—C10—C11	2.0 (10)	C14A—C9A—C10A—C11A	1.2 (9)
C8—C9—C10—C11	−180.0 (7)	C8A—C9A—C10A—C11A	−179.0 (7)
C14—C9—C10—C37	−177.8 (6)	C14A—C9A—C10A—C37A	−178.8 (6)
C8—C9—C10—C37	0.2 (12)	C8A—C9A—C10A—C37A	1.0 (12)
C9—C10—C11—C12	−0.9 (10)	C9A—C10A—C11A—C12A	0.8 (9)
C37—C10—C11—C12	178.9 (7)	C37A—C10A—C11A—C12A	−179.3 (6)
C10—C11—C12—C13	−0.3 (11)	C10A—C11A—C12A—C13A	−2.2 (10)
C11—C12—C13—C14	0.4 (10)	C11A—C12A—C13A—C14A	1.5 (10)
N2—N1—C14—C13	−179.2 (6)	N2A—N1A—C14A—C13A	−177.8 (6)
C7—N1—C14—C13	−13.2 (11)	C7A—N1A—C14A—C13A	−14.0 (11)
N2—N1—C14—C9	1.4 (7)	N2A—N1A—C14A—C9A	1.9 (7)
C7—N1—C14—C9	167.5 (6)	C7A—N1A—C14A—C9A	165.7 (6)
C12—C13—C14—N1	−178.5 (7)	C12A—C13A—C14A—N1A	−179.8 (6)
C12—C13—C14—C9	0.8 (9)	C12A—C13A—C14A—C9A	0.5 (9)
C10—C9—C14—N1	177.3 (6)	C10A—C9A—C14A—N1A	178.4 (6)
C8—C9—C14—N1	−1.2 (7)	C8A—C9A—C14A—N1A	−1.5 (7)
C10—C9—C14—C13	−2.1 (10)	C10A—C9A—C14A—C13A	−1.9 (10)
C8—C9—C14—C13	179.4 (6)	C8A—C9A—C14A—C13A	178.3 (6)
C3—C2—C15—C16	85.0 (7)	C1A—C2A—C15A—C16A	−96.2 (7)
C1—C2—C15—C16	−94.8 (7)	C3A—C2A—C15A—C16A	80.5 (7)
C24—N3—C17—C3	154.5 (6)	C24A—N3A—C17A—C3A	152.2 (6)
N4—N3—C17—C3	−25.7 (7)	N4A—N3A—C17A—C3A	−25.9 (7)
C2—C3—C17—N3	98.7 (6)	C4A—C3A—C17A—N3A	−78.8 (7)
C4—C3—C17—N3	−80.2 (7)	C2A—C3A—C17A—N3A	99.8 (6)
N3—N4—C18—C19	−0.3 (7)	N3A—N4A—C18A—C19A	−0.4 (6)
N4—C18—C19—C24	0.3 (7)	N4A—C18A—C19A—C20A	−178.7 (7)
N4—C18—C19—C20	−177.3 (6)	N4A—C18A—C19A—C24A	0.6 (7)
C24—C19—C20—C21	0.0 (9)	C24A—C19A—C20A—C21A	−0.1 (9)
C18—C19—C20—C21	177.2 (7)	C18A—C19A—C20A—C21A	179.1 (7)
C24—C19—C20—C38	−178.4 (6)	C24A—C19A—C20A—C38A	−179.8 (6)
C18—C19—C20—C38	−1.2 (11)	C18A—C19A—C20A—C38A	−0.6 (11)
C19—C20—C21—C22	0.6 (9)	C19A—C20A—C21A—C22A	0.6 (10)
C38—C20—C21—C22	179.0 (6)	C38A—C20A—C21A—C22A	−179.7 (7)
C20—C21—C22—C23	−0.5 (10)	C20A—C21A—C22A—C23A	0.0 (11)
C21—C22—C23—C24	−0.2 (9)	C21A—C22A—C23A—C24A	−1.0 (10)
N4—N3—C24—C23	178.4 (6)	N4A—N3A—C24A—C23A	−180.0 (6)
C17—N3—C24—C23	−1.9 (11)	C17A—N3A—C24A—C23A	1.9 (11)
N4—N3—C24—C19	−0.1 (7)	N4A—N3A—C24A—C19A	0.2 (7)
C17—N3—C24—C19	179.6 (5)	C17A—N3A—C24A—C19A	−178.0 (5)
C22—C23—C24—N3	−177.5 (6)	C22A—C23A—C24A—N3A	−178.4 (6)
C22—C23—C24—C19	0.8 (9)	C22A—C23A—C24A—C19A	1.4 (9)
C20—C19—C24—N3	177.9 (5)	C20A—C19A—C24A—N3A	179.0 (5)
C18—C19—C24—N3	−0.1 (6)	C18A—C19A—C24A—N3A	−0.4 (6)
C20—C19—C24—C23	−0.7 (9)	C20A—C19A—C24A—C23A	−0.9 (9)
C18—C19—C24—C23	−178.7 (6)	C18A—C19A—C24A—C23A	179.7 (6)
C5—C4—C25—C26	93.7 (7)	C5A—C4A—C25A—C26A	−91.4 (7)
C3—C4—C25—C26	−89.6 (7)	C3A—C4A—C25A—C26A	90.5 (7)
N6—N5—C27—C5	27.5 (9)	C34A—N5A—C27A—C5A	−149.0 (6)
C34—N5—C27—C5	−157.8 (6)	N6A—N5A—C27A—C5A	39.8 (8)
C4—C5—C27—N5	79.7 (8)	C4A—C5A—C27A—N5A	75.9 (7)
C6—C5—C27—N5	−100.9 (7)	C6A—C5A—C27A—N5A	−104.3 (7)
N5—N6—C28—C29	−1.0 (9)	N5A—N6A—C28A—C29A	−1.5 (7)
N6—C28—C29—C34	0.9 (9)	N6A—C28A—C29A—C34A	1.2 (7)
N6—C28—C29—C30	−179.3 (8)	N6A—C28A—C29A—C30A	−179.5 (7)
C34—C29—C30—C31	0.3 (10)	C34A—C29A—C30A—C31A	−0.1 (9)
C28—C29—C30—C31	−179.4 (8)	C28A—C29A—C30A—C31A	−179.4 (7)
C34—C29—C30—C39	179.7 (7)	C34A—C29A—C30A—C39A	179.3 (6)
C28—C29—C30—C39	0.0 (13)	C28A—C29A—C30A—C39A	0.0 (11)
C29—C30—C31—C32	2.4 (12)	C29A—C30A—C31A—C32A	1.6 (9)
C39—C30—C31—C32	−177.0 (8)	C39A—C30A—C31A—C32A	−177.8 (7)
C30—C31—C32—C33	−3.9 (13)	C30A—C31A—C32A—C33A	−1.7 (10)
C31—C32—C33—C34	2.3 (12)	C31A—C32A—C33A—C34A	0.1 (10)
N6—N5—C34—C29	−0.1 (8)	N6A—N5A—C34A—C29A	−0.6 (7)
C27—N5—C34—C29	−175.3 (6)	C27A—N5A—C34A—C29A	−172.6 (6)
N6—N5—C34—C33	−178.5 (8)	N6A—N5A—C34A—C33A	−178.8 (6)
C27—N5—C34—C33	6.3 (12)	C27A—N5A—C34A—C33A	9.3 (11)
C28—C29—C34—N5	−0.5 (8)	C30A—C29A—C34A—N5A	−179.8 (6)
C30—C29—C34—N5	179.7 (6)	C28A—C29A—C34A—N5A	−0.3 (7)
C28—C29—C34—C33	178.1 (7)	C30A—C29A—C34A—C33A	−1.5 (9)
C30—C29—C34—C33	−1.7 (11)	C28A—C29A—C34A—C33A	178.0 (6)
C32—C33—C34—N5	178.6 (7)	C32A—C33A—C34A—N5A	179.3 (6)
C32—C33—C34—C29	0.4 (11)	C32A—C33A—C34A—C29A	1.4 (9)
C1—C6—C35—C36	85.9 (7)	C5A—C6A—C35A—C36A	−92.4 (7)
C5—C6—C35—C36	−97.6 (7)	C1A—C6A—C35A—C36A	89.4 (7)

### Hydrogen-bond geometry (Å, º)

*Cg*1, *Cg*2 and *Cg*3 represent the centroids of the N3/N4/C18/C19/C24, N3*A*/N4*A*/C18*A*/C19*A*/C24*A* and N5*A*/N6*A*/C28*A*/C29*A*/C34*A* rings, respectively.

**Table:**

*D*—H···*A*	*D*—H	H···*A*	*D*···*A*	*D*—H···*A*
N7—H7*A*···N2^i^	0.91 (2)	2.02 (3)	2.923 (8)	171 (9)
N7—H7*B*···N4^i^	0.91 (2)	2.10 (4)	2.980 (8)	162 (8)
N7—H7*C*···N6^i^	0.91 (2)	2.01 (2)	2.923 (9)	175 (9)
N7—H7*D*···F3	0.91 (2)	2.06 (3)	2.951 (8)	164 (7)
N7—H7*D*···F5	0.91 (2)	2.54 (6)	3.213 (8)	131 (6)
C13—H13···F6*A*	0.95	2.55	3.500 (9)	176
C17—H17*A*···F1	0.99	2.52	3.156 (7)	122
C17—H17*B*···F6	0.99	2.60	3.179 (8)	117
C18—H18···F2^ii^	0.95	2.33	3.201 (8)	151
C22—H22···F5*A*^iii^	0.95	2.45	3.325 (8)	154
C23—H23···F4	0.95	2.60	3.552 (8)	175
C31—H31···F3^iv^	0.95	2.35	3.289 (8)	173
C37—H37*C*···F2*A*^ii^	0.98	2.51	3.379 (9)	148
N7*A*—H7*AA*···N2*A*^v^	0.91 (2)	2.02 (3)	2.917 (8)	171 (10)
N7*A*—H7*AB*···N4*A*^v^	0.91 (2)	2.09 (3)	2.969 (8)	161 (8)
N7*A*—H7*AC*···N6*A*^v^	0.92 (2)	2.03 (3)	2.941 (7)	172 (8)
N7*A*—H7*AD*···F3*A*	0.91 (2)	2.17 (3)	3.050 (7)	164 (6)
N7*A*—H7*AD*···F2*A*	0.91 (2)	2.43 (6)	3.096 (7)	130 (6)
C17*A*—H17*D*···F1*A*	0.99	2.59	3.247 (7)	124
C18*A*—H18*A*···F6*A*^vi^	0.95	2.44	3.317 (8)	154
C23*A*—H23*A*···F4*A*	0.95	2.61	3.561 (8)	177
C36—H36*B*···*Cg*2	0.98	2.77	3.481 (7)	130
C26*A*—H26*E*···*Cg*3	0.98	2.66	3.592 (7)	158
C26*A*—H26*F*···*Cg*2	0.98	2.56	3.506 (7)	163
C36*A*—H36*F*···*Cg*1^vii^	0.98	2.82	3.605 (7)	138

Symmetry codes: (i) *x*−1, *y*, *z*; (ii) *x*+1, *y*, *z*; (iii) −*x*+1, *y*−1/2, −*z*+1; (iv) *x*+1, *y*, *z*+1; (v) *x*, *y*, *z*−1; (vi) *x*, *y*, *z*+1; (vii) −*x*+1, *y*+1/2, −*z*+1.
